# A Case Report of Malfunctioning Peritoneal Dialysis Catheter in a Patient With Diverticulitis With a Phlegmon

**DOI:** 10.7759/cureus.14455

**Published:** 2021-04-13

**Authors:** Valerie Nwanji, Gail Burkholder, Gurwant Kaur

**Affiliations:** 1 Internal Medicine, Penn State Health Milton S. Hershey Medical Center, Hershey, USA; 2 Medicine/Nephrology, Penn State Health Milton S. Hershey Medical Center, Hershey, USA

**Keywords:** peritoneal dialysis, diverticulosis, diverticulitis, peritonitis, peritoneal dialysis catheter, kidney transplant, phlegmon, focal segmental glomerulosclerosis, chronic kidney disease, end stage renal disease

## Abstract

We are presenting a case of 59-year-old female with advanced chronic kidney disease where her peritoneal dialysis (PD) catheter was complicated and malfunctioned by its entanglement within a phlegmon from diverticulitis. It needed removal of PD catheter and eventually sigmoid colectomy. We reviewed the literature regarding the risk of peritonitis in patients with asymptomatic diverticulosis and in symptomatic patients with diverticulitis.

## Introduction

A 59-year-old woman with end-stage renal disease (ESRD), who was started on continuous cyclic peritoneal dialysis (CCPD) in February of 2017. Approximately 24 years prior to this, she was diagnosed with chronic kidney disease (CKD) secondary to focal segmental glomerulosclerosis (FSGS) by a native kidney biopsy. She eventually progressed to develop ESRD, requiring peritoneal dialysis (PD). She had a Missouri swan neck (coiled) PD catheter placed on the left side of her abdomen. As per the operative report, the entry-site was marked at the first cuff. The exit-site was about 3-4 cm from the second cuff.

She was able to tolerate and manage CCPD with assistance from her husband. As per her outpatient PD nurse, she had difficulties draining the fluid and complained of abdominal discomfort. She was tried on tidal PD (85%) to ease her abdominal discomfort on draining. However, it didn’t provide her much relief. She had regular bowel movements and no laxatives were required. She had no episodes of exit-site infection or peritonitis as per clinic records. She was able to come off PD after approximately eight months; she was taken off PD about one week before this presentation due to improved kidney function with an estimated glomerular filtration rate (eGFR) of 20 ml/min/1.73 m^2^. Before presentation, she was not able to flush (fill in or drain) her PD catheter at home; and then she presented to the emergency department with abdominal pain. She was a former smoker and has recently stopped working due to her health issues. 

Three days prior to presentation, she began to have suprapubic discomfort, which then became diffuse abdominal pain. She developed intermittent fevers in a span of two days, with a maximum temperature of 101°F. She had no associated nausea, vomiting, diarrhea, or constipation. She was admitted under colorectal surgery for diverticulitis. The nephrology team was consulted to evaluate during her inpatient stay.

## Case presentation

**Figure 1:**
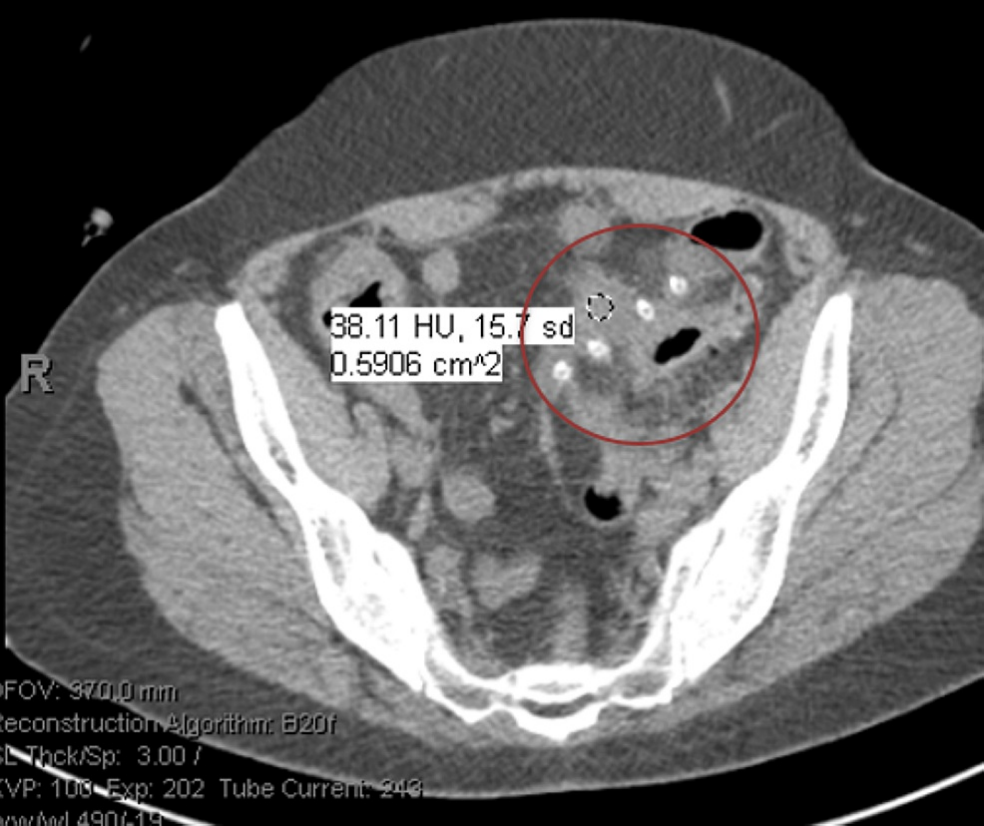
Computed tomography (CT) abdomen/pelvis without contrast showing phlegmon from acute diverticulitis. It was also noted that the peritoneal dialysis catheter terminated in the left lower quadrant with tip adjacent to inflammatory changes and the phlegmon (in red circle).

**Figure 2:**
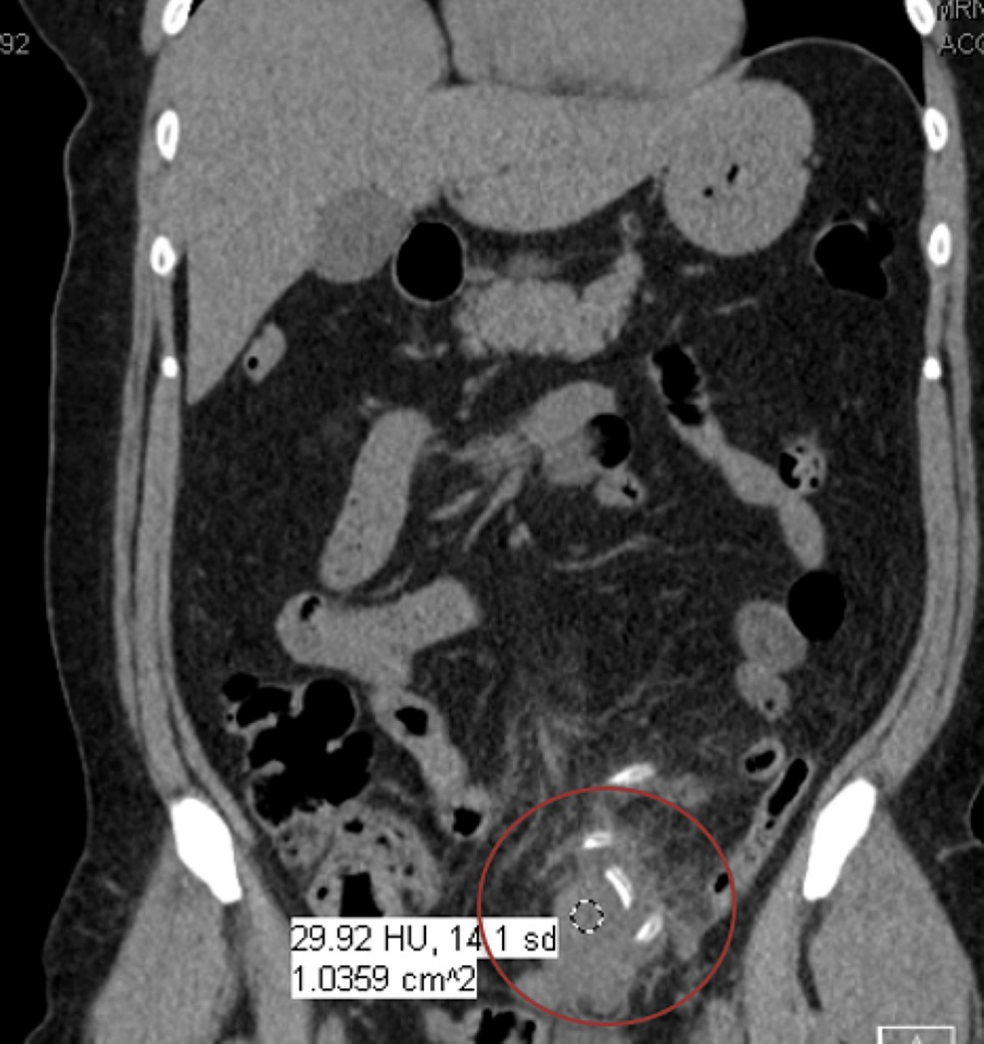
Computed tomography (CT) abdomen/pelvis without contrast showing peritoneal catheter was wrapped within the omentum and phlegmon (in red circle).

During presentation, she was not in acute distress, her abdominal exam showed localized tenderness over the left lower quadrant. In the emergency room, small aspirate from PD catheter was obtained by emergency nurse staff, and was sent for cell count and fluid culture. Her details of PD prescription before admission are listed in Table 1. Aspirate from the peritoneal catheter grew *Staphylococcus epidermidis*. Her initial blood work is listed in Table 2 and Table 3. In the lab, aspirate was diluted to run a cell count and results were not reliable (Table 4). During the early hospital course, one of two blood cultures grew *Staphylococcus epidermidis* as well, but repeat cultures showed no growth. CT abdomen/pelvis without contrast was obtained and showed acute sigmoid diverticulitis with an associated phlegmon, small amount of free fluid, and no free air. It was noted that the peritoneal dialysis catheter terminated in the left lower quadrant with a tip adjacent to inflammatory changes and the phlegmon (Figures 1, 2). Peritoneal catheter was wrapped within the omentum and phlegmon. She was initially given Vancomycin, and then was treated with ciprofloxacin, metronidazole, and linezolid for diverticulitis for 14 days.

**Table 1:** Details of peritoneal dialysis (PD) prescription.

Home PD prescription
Fill volumes: 1,700 millilitres (mL) (tidal 85%)
No. of exchanges: 4
Dextrose solution: alternating 1.5% and 2.5%
Average ultra-filtration: 500 mL
Average dwell time: 1 hour and 38 minutes

**Table 2:** Complete blood count.

Parameter	Value
White blood cells	11.08 thousand cells/microliter (k/uL)
Hemoglobin	11.3 grams/deciliter (g/dL)
Hematocrit	33.9%
Platelet count	260 k/uL

**Table 3:** Serum chemistry results. GFR: glomerular filtration rate.

Parameter	Value
Sodium	138 millimole/liter (mmol/L)
Potassium	5.0 mmol/L
Chloride	101 mmol/L
Bicarbonate	23 mmol/L
Blood urea nitrogen	44 milligrams/deciliter (mg/dL)
Glucose	91 mg/dL
Calcium	9.0 mg/dL
Phosphorus	3.2 mg/dL
Estimated GFR	17 mL/minute/1.73 meter^2^
Phosphorus	3.2 mg/dL
Estimated GFR	17 mL/min/1.73 m^2^

**Table 4:** Dialysate fluid analysis*. *It is to be noted that peritoneal dialysate/fluid analysis was done on peritoneal dialysis catheter aspirate; it was diluted to run cell count and was not reliable.

Parameter	Value
Nucleated cells	18 cells/microlitre (cells/uL)
Red blood cells	<1,000 cells/uL
Neutrophils	98%
Lymphocyte	2%

She showed improvement in her clinical symptoms with medical management. It was decided to remove her PD catheter, because of improved renal function and improvement of diverticulitis. PD catheter was removed approximately three months after her presentation on outpatient basis. She was recommended to undergo a sigmoid resection especially when she was considering the possibility of a kidney transplant in the future in lieu of risk of infection with post-transplant immunosuppression. She underwent a laparoscopic sigmoid colectomy about 17 months after her initial presentation. The sigmoid colectomy procedure and the subsequent hospital course were uncomplicated. She has not required dialysis and remained free of uremic symptoms. Due to improvement of her clinical status, and no sign of infection, she was made active on the kidney transplant list again. She received a deceased donor renal transplant in March of 2020.

## Discussion

Currently, there is no universally accepted standard of clinical practice for the management of diverticulosis and acute diverticulitis while on PD. This is likely from limited individual center experiences and conflicting literature data in regards to performing PD in diverticular disease and the risk of peritonitis in such patients. Currently, individual center judgement drives the management decision. It is possible that surgery, nephrology and infectious disease teams could not agree on a plan of management in peritonitis associated with diverticulitis. Our case is unique in that the decision to discontinue PD was made a week before the diagnosis of diverticulitis. Although there were early signs of trouble by way of poor PD catheter function prior to stopping the PD.

There remains a clinical dilemma regarding the removal of PD catheter, whether catheter should be removed in all and at an early stage in peritonitis due to diverticulitis. Medical management has a role in the management of such patients along with collaboration with surgeons. It is clear, should the PD catheter get entrapped in the phlegmon of diverticular infected mass, interfering with the performance of peritoneal dialysis, surgery should be performed early. Removing the phlegmon at the same time would be a decision made by the surgeon on the table depending upon the nature and extent of the disease. If the catheter is not involved, surgical approach is usually elective. Upon clearing the infection, electively phlegmon removal with colostomy/sigmoid removal can be performed. However, should peritonitis become refractory, surgical intervention would become necessary. PD catheter removal was made easy because of kidney function recovery at the time of acute diverticulitis. We don’t have a clear answer if delaying the surgical approach in the early clinical course would have any damaging effects on the peritoneal membrane permanently and if would jeopardize the patient’s future chances of continuing PD. Contrary to surgical approach, a recent case report of endogenous peritonitis from an acute appendicitis was interesting and unusual. In this case, PD catheter was salvaged, despite the surgical removal of appendicitis [1].

In order to understand the risk of diverticulitis in PD patients with diverticulosis, Toda and co-workers from Japan suggested that radiological findings of sigmoid diverticula were not identified as risk factors for diverticulitis and/or peritonitis [2]. They also recommended that PD may be contraindicated in patients who are presenting with frequent episodes of lower abdominal pain. Interestingly, a Chinese study, found that sigmoid diverticula were found more frequently in Western world patients versus Chinese patients who predominantly demonstrate diverticulosis on the right side and are at increased risk of peritonitis [3]. It is not clear the significance of this interesting observation. A study in 129 patients on continuous ambulatory peritoneal dialysis (CAPD) in Sweden suggests that ≥ 10 diverticula, ≥ 10 mm in size and the presence of diverticula in the ascending, transverse or descending colon significantly increased the risk of peritonitis of enteric origin [4]. CT scan with or without the contrast is useful in detecting diverticulosis and diverticulitis [2].

In our patient, the growth of *Staphylococcus epidermidis* from dialysate and blood culture is likely from contamination; otherwise difficult to explain in the presence of diverticulitis. A thought-provoking editorial by the legendary Dr. Oreopoulos suggested that elderly PD patients can easily be predisposed to develop fecal peritonitis from micro-perforations in milder, non-symptomatic cases of diverticulitis. This could be explained by the fact of continuous presence of dialysis solution in the peritoneal cavity in PD patient could arguably interfere with the normal healing process, initiated by the lymphatics in the omental tissue. This is the first step in the host defense mechanism of the peritoneal cavity on the initiation of infection/inflammation [5]. On the contrary, in the general population, such low-grade diverticulitis resulting in micro-perforations could heal without resulting in a major problem, or could be passed off as a non-significant event. The host-defense function of omental tissue repairs the milder diverticular infectious event [5]. This sheds light on the risk of diverticulitis in PD patients might be different than seen in the general population. He further suggested that such peritonitis usually do not respond to medical management, definitive treatment usually requires peritoneal catheter removal.

On the contrary, expert opinion suggests, peritoneal endogenous defense can help take care of milder cases of diverticular infections, especially if they are small and CAPD can be interrupted for 1-3 weeks [5]. Very intuitive paper by Setyapranata and Holt describes how gut and peritoneal dialysis solutions interact with each other [6]. Presence of PD solutions affects gut mobility and function. It can increase the risk of hernia, urinary incontinence especially in elderly patients on PD. The bowel loops in the peritoneal cavity in pool of dialysis solution could interfere with the successful performance of PD, e.g., frequent occurrence of constipation due to phosphate binders could increase the risk of peritonitis promoting transmural migration of bacteria, etc. [7].

From the review of literature, it is clear that there is no factual information that could aid in evidence-based management of diverticulosis/diverticulitis in patients on PD. More observations and studies are needed to clarify these special group of patients, especially the elderly who routinely undergo PD without addressing the complex risk factors associated with diverticular diseases in PD patients.

## Conclusions

Due to the lack of evidence-based information, decisions to initiate and maintain PD in patients with extensive diverticulosis should be made after discussing the clinical and imaging data. Individual case should be accepted for PD, based on the expertise of physician and dialysis center along with prior symptomatology of patients. However, the presence of diverticular disease should not automatically preclude the option for PD. Our patient had enough recovery of her renal function to be able to come off PD. She underwent sigmoid colectomy after the episode of peritonitis-related acute diverticulitis. We presume, because of the diverticular complication she endured, she might not have been able to successfully perform PD, when dialysis would have become necessary. Hemodialysis would have been another option for her. However, we are glad to share the fact that she has successfully received a kidney transplant without having to undergo dialysis in the interval times. 
